# The Drinkers’ Intervention to Prevent Tuberculosis (DIPT) trial among heavy drinkers living with HIV in Uganda: study protocol of a 2×2 factorial trial

**DOI:** 10.1186/s13063-021-05304-7

**Published:** 2021-05-20

**Authors:** Sara Lodi, Nneka I. Emenyonu, Kara Marson, Dalsone Kwarisiima, Robin Fatch, Michael G. McDonell, Debbie M. Cheng, Harsha Thirumurthy, Monica Gandhi, Carol S. Camlin, Winnie R. Muyindike, Judith A. Hahn, Gabriel Chamie

**Affiliations:** 1 Department of Biostatistics, Boston University School of Public Health, 801 Massachusetts Avenue, Boston, MA 02118 USA; 2 Division of HIV, Infectious Disease and Global Medicine, University of California San Francisco, San Francisco, USA; 3 Infectious Diseases Research Collaboration, Kampala, Uganda; 4 Elson S. Floyd College of Medicine, Washington State University, Spokane, USA; 5 Perelman School of Medicine at the University of Pennsylvania, Philadelphia, USA; 6 Department of Obstetrics, Gynecology & Reproductive Sciences, University of California San Francisco, San Francisco, USA; 7 Global Health Collaborative, Mbarara University of Science and Technology, Mbarara, Uganda; 8 Mbarara Regional Referral Hospital, Mbarara, Uganda

**Keywords:** Tuberculosis, Alcohol drinking, HIV, Isoniazid preventive therapy, Contingency management, Urine ethyl glucuronide, Isoscreen, Adherence, Point of care, Incentives, Phosphatidylethanol, Randomized controlled trial

## Abstract

**Background:**

The risk of tuberculosis (TB) is high among *p*eople *w*ith *H*IV (PWH). Heavy alcohol drinking independently increases TB risk and approximately 25% of PWH globally engage in heavy drinking. While isoniazid (INH) preventive therapy decreases TB incidence and mortality among PWH, heavy drinking during INH is associated with liver toxicity and poor adherence. Interventions are, therefore, urgently needed to decrease alcohol use and improve adherence to INH in this population in settings with high prevalence of HIV and TB like Uganda.

**Methods:**

The Drinkers’ Intervention to Prevent TB (DIPT) study is a 2×2 factorial randomized controlled trial among HIV/TB co-infected adults (≥18 years) who engage in heavy alcohol drinking and live in Uganda. The trial will allocate 680 participants with a 1:1:1:1 individual randomization to receive 6 months of INH and one of the following interventions: (1) no incentives (control), (2) financial incentives contingent on low alcohol use, (3) financial incentives contingent on high adherence to INH, and (4) escalating financial incentives for both decreasing alcohol use and increasing adherence to INH. Incentives will be in the form of escalating lottery-based monetary rewards. Participants will attend monthly visits to refill isoniazid medications, undergo liver toxicity monitoring, and, except for controls, determine eligibility for prizes. We will estimate (a) the effect of incentives contingent on low alcohol use on reduction in heavy drinking, measured via a long-term objective and self-reported metric of alcohol use, at 3- and 6-month study visits, and (b) the effect of incentives contingent on high adherence to INH, measured as >90% pill-taking days by medication event monitoring system cap opening. We will use qualitative methods to explore the mechanisms of any influence of financial incentives on HIV virologic suppression.

**Discussion:**

This study will provide new information on low-cost strategies to both reduce alcohol use and increase INH adherence among people with HIV and TB infection who engage in heavy drinking in low-income countries with high HIV and TB prevalence.

**Trial registration:**

ClinicalTrials.gov NCT03492216. Registered on April 10, 2018

## Introduction

### Background and rationale

Tuberculosis (TB) is the leading cause of death among *p*eople *w*ith *H*IV (PWH) worldwide, accounting for 251,000 deaths in 2018 (33% of HIV-associated deaths) [1]. In addition, the risk of active TB disease among individuals with latent TB infection is increased 3-fold among people who engage in heavy drinking compared to non-drinkers [2–6]. Globally, an estimated 25% of PWH engage in heavy drinking and the prevalence of heavy drinking is higher in PWH than in the general population [7, 8]. Thus, PWH who drink heavily are a vulnerable population at very high risk of morbidity and mortality due to TB disease.

Isoniazid (INH) preventive treatment (IPT) combined with antiretroviral treatment is currently the most well-established strategy to prevent active TB among PWH with latent TB infection [9–12]. However, the use of INH among persons who engage in heavy drinking is problematic due to the increased risk of hepatotoxicity from dual injury. Most INH-associated hepatoxicity is reversible by stopping therapy, but liver injury can lead to liver failure [13]. As a result, in many settings with a high prevalence of TB and HIV where routine monitoring for liver toxicity is challenging, such as in Uganda, national guidelines list heavy alcohol use as a contraindication for INH use [14]. Furthermore, heavy alcohol use has been shown to reduce adherence to medications, including antiretroviral treatment [15–18], and there is evidence of high rates of INH discontinuation in persons who engage in heavy drinking [19–24]. Thus, interventions are needed to both decrease alcohol use and increase INH adherence in this high-risk population, which together would lead to reductions in INH toxicity, TB morbidity, and mortality.

The use of incentives, in the form of “contingency management” strategies that provide monetary rewards (cash, vouchers, etc.) contingent on the achievement of desired behaviors, has been shown to be highly effective for reducing substance use, including alcohol use [25, 26]. There is also some evidence that incentive-based interventions can be used to improve medication adherence [27, 28]. These approaches, which are motivated by theoretical insights from economics and psychology, provide a rationale for offering financial incentives to individuals for undertaking a targeted behavior, e.g., alcohol abstinence or medication adherence. Such incentive strategies may create a window for safe and effective INH use by decreasing hepatotoxicity and increasing INH adherence. However, the potential benefits of financial incentives to reduce alcohol use or to increase INH adherence among persons co-infected with HIV and TB in resource-limited settings have not been explored to date due to the previous lack of rapid, objective, and reliable tests to monitor these behaviors. These tests are now available.

Recent technological advances allow for point-of-care (POC) urine testing to measure alcohol use with an ethyl glucuronide (EtG) dipstick. The test provides a positive result up to 3 days after heavy drinking [29, 30]. Additionally, the INH IsoScreen urine test can test for INH use in the last 24 h [31]. These two tests create an opportunity to test incentive-based interventions using objective and rapid metrics during INH therapy among PWH who engage in heavy drinking.

Here we describe the protocol of the Drinkers’ Intervention to Prevent TB (DIPT) randomized controlled trial which is designed to determine if incentive-based approaches can reduce alcohol use and improve medication adherence (measured objectively) to INH in PWH co-infected with TB who engage in heavy drinking in Uganda. This study will provide new information on low-cost strategies to both reduce alcohol use and increase INH adherence during 6 months of IPT in low-income countries.

### Trial design and setting

DIPT is a randomized, 2×2 factorial trial among adults co-infected with HIV/TB and who are heavy alcohol users in southwestern Uganda. This region has an HIV prevalence of 8% [32], and TB infection prevalence is estimated at 28–50% [33, 34]. The study will be conducted at participating clinics in an urban setting (Mbarara town) and two rural settings (Rugazi and Ruhoko).

**Fig. 1:**
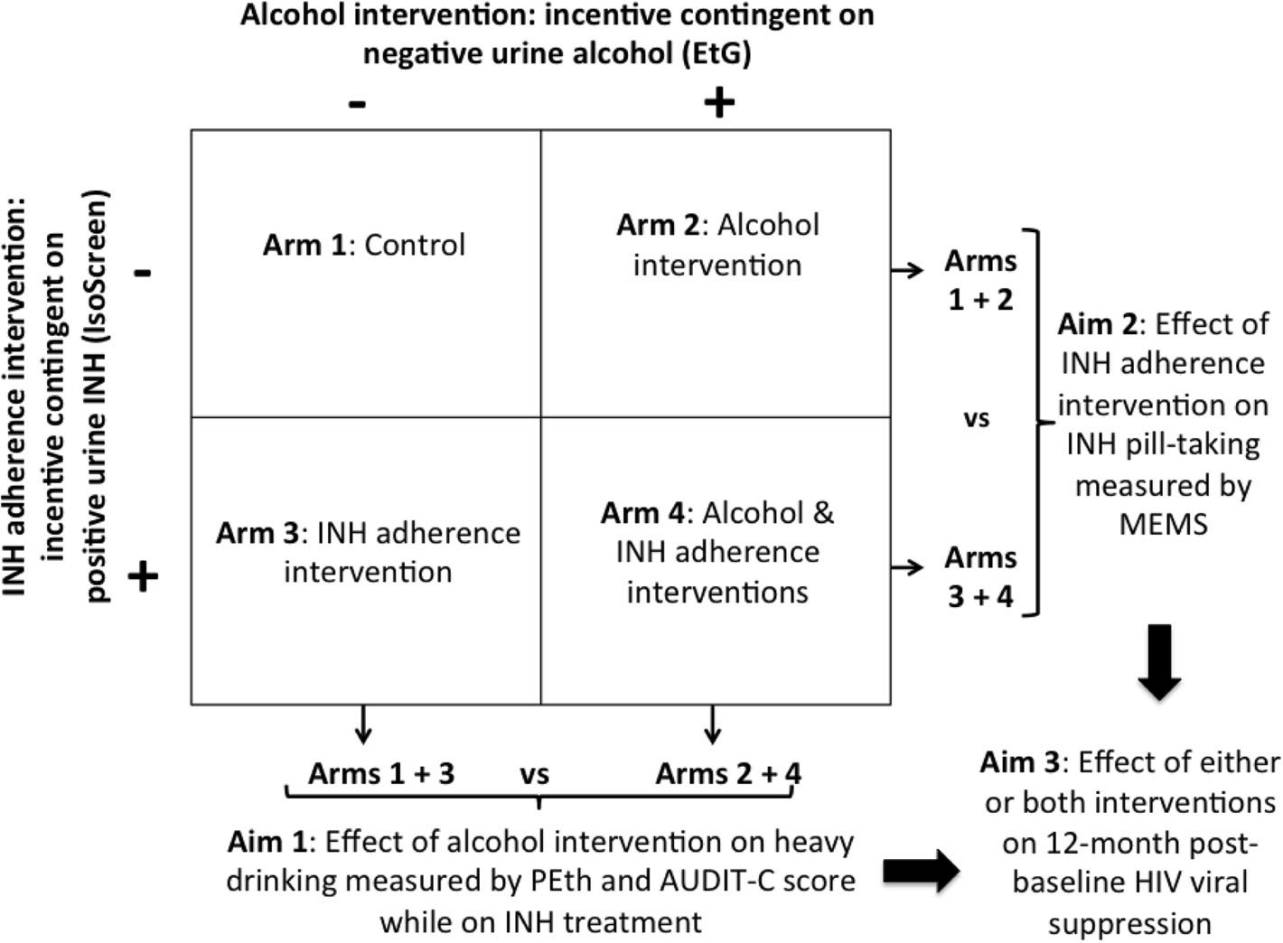
Schematic of study design and aims of the Drinkers’ Intervention to Prevent TB (DIPT) trial

Figure 1 summarizes the study design of the trial. Eligible participants receive 6 months of INH and are randomly allocated to one of the following four arms: *Arm 1*: no incentives (control), *Arm 2*: financial incentives contingent on low alcohol use at INH refill visits, *Arm 3*: financial incentives contingent on high INH adherence at INH refill visits, and *Arm 4*: financial incentives contingent on decreasing alcohol use and on increasing INH adherence (rewarded independently). Low alcohol use is measured via a negative POC ethyl glucuronide (EtG) urine test (EtG is a metabolite of alcohol use that can be detected in urine for up to 2 to 3 days after heavy drinking [31]) defined as EtG<300 ng/mL. High adherence to INH is measured via a positive POC IsoScreen test, a marker for INH ingestion in the prior 24 h [35, 36].

### Objectives

The DIPT trial is designed to achieve two aims. *Aim 1*: To determine the effectiveness of financial incentives contingent on low alcohol use (Arms 2 and 4) versus no alcohol incentives (Arms 1 and 3) to reduce heavy drinking during the course of INH treatment. The primary hypothesis for Aim 1 is that participants receiving incentives for negative urine EtG testing will have lower alcohol use, determined via a medium-term alcohol biomarker during INH, compared to participants receiving no alcohol incentives. *Aim 2*: To determine the effectiveness of financial incentives contingent on high INH adherence (Arms 3 and 4) versus no INH adherence incentives (Arms 1 and 2) to increase INH adherence during a 6-month IPT course. The primary hypothesis for Aim 2 is that participants receiving INH adherence incentives will have better adherence to INH, determined via objective monitoring, than participants receiving no INH adherence incentives.

## Methods: participants, interventions, and outcomes

### Recruitment and eligibility criteria

Participants who report any alcohol use at clinic visits for HIV care are recruited from the participating clinics. In all sites of enrollment, a clinic staff member, who is also a member of the research team, solicits participation of persons with HIV aged ≥ 18 years, who report alcohol use, have been on ART for at least 6 months, and have no previous history of TB, TB treatment, or TB preventative therapy. In addition, potential participants must be fluent in Runyankole or English (the two most widely spoken languages in the area), be living within a 2-h driving distance or 60 km of the study site, and have no plans to move out of the catchment area within 6 months. These individuals will be invited to take part in the screening process. On the same day, they provide a urine test and a blood sample; they are screened for active TB disease based on the WHO 4-symptoms screen assessment [37]; and they receive a tuberculin skin test (TST) for latent TB infection. Patients are then asked to return in 2 days for the TST reading.

Eligible participants for the trial are those who (1) self-report current heavy alcohol use defined by the Alcohol Use Disorder Identifier Test-Consumption (AUDIT-C) positivity (≥3 for women, ≥4 for men) for the prior 3 months and have a positive urine EtG test, (2) have latent TB infection defined as a positive TST test (≥5 mm induration), and (3) have normal liver enzymes defined as aspartate aminotransferase [AST] and alanine aminotransferase [ALT] less than twice the upper limit of normal.

Ineligible participants are women with a same day positive pregnancy test result (given evidence of increased risk of adverse pregnancy outcomes among women living with HIV taking INH during pregnancy compared to women living with HIV who initiated INH post-partum [38]), individuals who were prescribed anti-convulsion medications or with history of recurring seizures (in accordance with Uganda Ministry of Health guidelines, given concerns for INH resulting in reduced seizure threshold, a rare adverse effect [39]), and individuals whose gross inebriation prevented them from providing informed consent. The study will also exclude individuals who were prescribed nevirapine in the past 3 months due to the hepatoxicity of this antiretroviral drug and those who started dolutegravir in the past 3 months, in accordance with Uganda Ministry of Health guidelines. Individuals who are not eligible for the study may return to be screened again after 3 months.

### Ethics and consent

When initially approached by the study staff, potential participants are given an introduction to the purpose of the study and the reasons that they have been approached for participation. There are separate consent forms for the screening procedures and for participating in the trial once eligibility is established. Informed consent documents are available in English and the local dialect (Runyankole). Participants will be informed of their right to not enroll and to withdraw from the study at any time. The potential risks associated with the study include risks due to INH, loss of confidentiality, stress from the study interview, and risks associated with blood draws. On the consent form, participants will be informed that only members of the research study team will have access to personal data collected during the trial and if information from the study is published or presented at scientific meetings, participant names or other personal identifying information will not be used. Each participant will be assigned a unique trial identifier, which will be used on all research documents to protect his/her identity and to ensure anonymity for the statistical analyses.

## Intervention description

Immediately after randomization, all participants will initiate a 6-month course of INH and will receive pyridoxine (B6), as well as brief alcohol and adherence counseling according to the Ugandan Ministry of Health guidelines [40]. INH will be dispensed in pill bottles with medication event monitoring system caps. Participants randomized to the three financial incentive arms will provide a urine sample for the study arm-appropriate testing at each refill visit. Participants randomized to alcohol reduction incentives (Arms 2 and 4) who have a negative urine EtG test will instantly win cash prizes by drawing one or more lottery scratch cards. The lottery scratch card will reveal low-, medium-, and high-value cash prizes. The number of lottery scratch cards awarded per participant will increase by one card at each subsequent visit with a negative urine EtG result, thereby providing an escalating prize incentive for sustained reductions in alcohol use. If a participant has a positive urine EtG test, no lottery scratch cards will be given, and the participant will “reset” to drawing one lottery card if the urine is EtG negative at the subsequent visit. Escalating incentives with a “reset” were chosen to offer greater reward for continuous, rather than intermittent, reductions in alcohol use during INH and to elicit loss aversion (i.e., fear of losing what one has gained) [41–43].

Similarly, participants randomized to receive INH adherence incentives (Arms 3 and 4) who have a positive urine IsoScreen test will win a prize by drawing one or more lottery cards using an escalating prize incentive design identical to the incentives for decreasing alcohol use. Participants randomized to receive both interventions (Arm 4) will be eligible to receive lottery card draws separately for each EtG and IsoScreen test and cash prizes will be awarded independently.

Cash prize values were chosen during a preparatory phase in consultation with community members and clinic staff at each site. Prize values that are too low might be insufficient to reinforce reductions in heavy drinking or adherence to INH, while prize values that are too high might decrease generalizability to real-world settings. We will limit low-value prizes to no more than US $5, representative of 1 week’s worth of wages in rural Uganda [44]. We will limit high-value prizes to no more than 10 times the low-value amount (US $50). Medium prizes will range in value between the low- and high-value amount. All lottery cards will have at least a low-value prize and the probabilities of winning will range from 1 to 5% for high-value prizes and 5 to 10% for medium-value prizes. If a participant misses a monthly visit, the incentive scheme will reset to baseline (drawing one lottery card only if the incentive condition is met).

Participants who are recommended to stop INH treatment due to toxicity or pregnancy will remain in the study following treatment discontinuation for follow-up monitoring. They will no longer be eligible to receive incentives for positive IsoScreen urine tests if they are in study arms 3 and 4, but will still be eligible to receive incentives from EtG testing if they are in study arms 2 or 4.

All participants will remain on their antiretroviral treatment medication or other concomitant medications for other comorbidities during the study period.

## Participant timeline and procedures

**Fig. 2:**
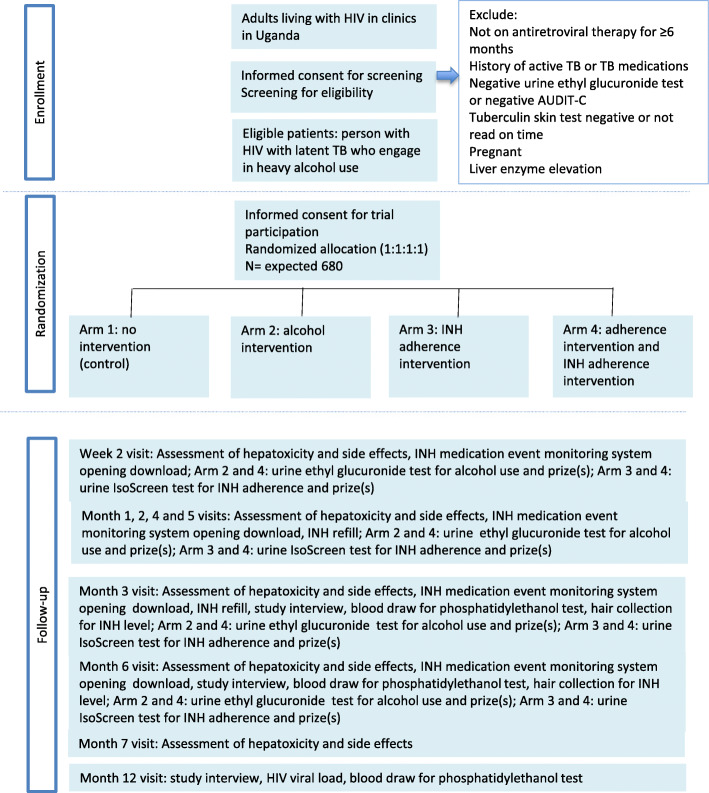
Schedule of enrolment, interventions, and assessments

Figure 2 illustrates the participant flow throughout the trial. Table 1 summarizes the data collected at study visits. After a participant is enrolled in the trial and consented, a 45-min interviewer-administered survey and a blood draw will occur. The survey is used to assess participant characteristics including demographics, mental and physical health, alcohol use, and self-reported adherence to antiretroviral therapy (ART). The blood draw is used to measure phosphatidylethanol (PEth), CD4 count, and HIV viral load. These will be used to describe the study population and to assess potential post-randomization imbalance (Table 1). PEth is extracted from dried blood spots and levels measured using LC/MS-MS for the 16:0/18:1 homologue [58]. HIV viral load is measured using a Cepheid Xpert HIV-1 RNA assay run on an existing GeneXpert platform in Mbarara, Uganda. This assay has been validated relative to the Abbott RealTime HIV-1 assay.

**Table 1:** Description of patient characteristics collected at baseline and at follow-up visits

Domain	Time of measurement	Individual variables
Demographics	Baseline	Age, sex, religion, household assets [45], and literacy
Health	Baseline	Medical Outcomes Study-HIV scale for quality of life and overall physical and mental health functioning [46–50], body mass index, CD4 cell count, active hepatitis B viral infection, smoking, and duration on ART, age first alcohol use, diagnostic and statistical manual (DSM)-5 alcohol criteria [51]
Alcohol use	Baseline, 3-, 6-, and 12-month visits	AUDIT, AUDIT-C, drinking patterns (days of the week), drinking locations, companions, and days of the week, frequency of alcohol intoxication, type of alcohol, 30-day timeline follow-back [52], biological measure (phosphatidylethanol (PEth))
INH adherence	Each refill visit	Number of MEMS openings, hair collection for INH level (at 3- and 6-month visits only)
INH side effects	Each refill visit	AST and ALT testing
ART adherence	Baseline, 3, 6, and 12 months	ART adherence Single Item Rating Scale [53, 54], visual analog scale, viral suppression
Psychosocial scales	Baseline	Center for Epidemiologic Studies Depression Scale (16 items) [55], Stages of Change Treatment Eagerness Scale (19 items) [56]
Discount rate (high vs. low)	Baseline	Time preferences (a measure of present bias and temporal discounting, i.e., tendencies towards instant versus delayed gratification) [57]

Visits for INH refills will occur at week 2 and at months 1 to 6 for all study participants (Fig. 2). During these visits, the participants will be assessed for liver toxicity, through screening for side effects and same day liver enzyme testing (ALT and AST). Urine samples will be collected and prizes will be distributed according to the goals of the participants’ intervention group. A urine pregnancy test will be given at the refill follow-up visits to all women of childbearing age who report they might be pregnant. All participants will be assessed for active TB based on the WHO 4-symptom screen at each visit, with further testing for those with positive symptoms. Those diagnosed with active TB will be offered TB treatment at their local TB clinic and disenrolled from the trial.

Follow-up structured interviews assessing alcohol use (AUDIT-C) and other covariates and secondary outcomes, and blood collection for PEth testing will occur at the 3-, 6-, and 12-month visits. HIV viral load will be measured at 6 and 12 months. Hair samples will be collected at 3- and 6-month visits for all participants to measure long-term INH concentrations [59]. Adherence will be measured at all refill visits through assessing bottle openings using a medication event monitoring system (MEMS).

## Retention

Follow-up phone calls, home visits when needed, and reimbursement for travel expenses of up to $5 for attending the study visits will be used to promote high retention for participants in all study arms, including controls.

## Primary outcomes

The study has two primary outcomes, one for each aim. The primary outcome for Aim 1 (i.e., to estimate the effect of incentives contingent on negative urine EtG tests) will be no heavy alcohol consumption over the 6 months of INH preventive therapy. This will be measured as a composite outcome of both PEth <35 ng/mL and negative AUDIT-C score at both the 3- and 6-month visits. PEth is a highly specific long-term marker for alcohol consumption that remains positive several weeks after heavy drinking. The AUDIT-C score, a measure of heavy drinking based on self-report, comprises a range for typical numbers of drinks and frequency of heavy drinking. For this study, we will modify the AUDIT-C score to cover the prior 3 months as opposed to the past 12 months (i.e., the window used in the original AUDIT-C score definition) to better capture current drinking [60, 61]. We will use standard cutoffs (≥3 for women and ≥4 for men) to define heavy drinking.

The primary outcome for Aim 2 (i.e., to estimate the effect of incentives contingent on a positive IsoScreen test) will be INH adherence measured as >90% pill-taking days by MEMS cap opening during the 6-month course of INH. MEMS adherence will be calculated as the number of pill bottle openings (no more than 1 per day counted) divided by the number of prescribed doses (180, unless the participant discontinued the medication).

## Secondary outcomes

We will examine other measures of heavy drinking as secondary outcomes for Aim 1 including self-reported number of drinking days in the prior 30 days, number of heavy drinking days (defined as ≥4 drinks/occasion and ≥5 drinks/occasion for women and men, respectively), and PEth as a continuous variable.

We will examine other measures of INH adherence as secondary outcomes for Aim 2 including drug concentration (ng/mg) in small hair samples at 3 and 6 months and MEMS-measured adherence as a continuous variable. Hair levels of INH measure long-term and cumulative exposure to INH and can be used to determine if short-term increases in adherence translate to sustained changes in behavior [62].

Another secondary outcome will be treatment discontinuation due to grade 3 or grade 4 hepatoxicity at any time while receiving INH. Grade 3/4 hepatoxicity will be defined as ALT or AST>3–5 times the upper limit of normal (ULN) and symptoms (nausea, vomiting, jaundice, or fatigue) or ALT and AST>5× ULN regardless of symptoms [11].

We will estimate the impact of financial incentives on HIV virologic suppression defined as an undetectable plasma HIV viral load at the 12-month visit and active TB during the 12 months of follow-up. Active TB will be defined as confirmed (if Xpert MTB/RIF assay positive) or suspected (based on chest x-ray findings or response to anti-TB treatment in a symptomatic and Xpert assay negative person).

## Sample size

With 680 enrolled participants (170 participants per arm), assuming 10% loss to follow-up, we anticipate that 612 participants will complete both the 3- and 6-month visits. For a 2-sided chi-square test with continuity correction and a significance level of 0.05, this sample size gives our study an 80% power to detect a 10% absolute difference in proportions with no heavy drinking between participants randomized to alcohol incentives (Arms 2 and 4) versus no alcohol incentive (Arms 1 and 3), assuming proportions of no heavy drinking of 25% and 15% for alcohol incentives and non no alcohol incentives, respectively.

Based on previous studies [24, 63], we expect poor completion of the INH course among PWH with heavy alcohol drinking, i.e., 62% vs. 50% in the INH adherence incentive group (Arms 1 and 4) and in the no INH adherence incentive group (Arms 2 and 3), respectively. Based on these assumptions, the study has 80% power to detect an absolute difference of 12% in the proportions with >90% of INH pills consumed using a chi-square test with continuity correction.

## Random allocation

After eligibility is determined and baseline assessments and blood draws are completed, participants are enrolled and randomized to one of the four intervention groups. Randomization is 1:1:1:1, stratified by gender and by study site. Permuted blocks with random block sizes of 4 and 8 are used to ensure balance with respect to the number of participants in each arm while making treatment assignment completely unpredictable [64]. To engage participants during the randomization process and provide transparency, we use pre-printed scratch cards revealing the randomization arm when scratched by a participant, after the participant draws their scratch card from a bag containing multiple cards.

## Blinding and limitation of risk of bias

This is an open-label study, as it is impossible to blind participants or research assistants to the study arm due to the nature of the interventions and financial rewards. However, the study arm is not revealed to clinic counselors or clinicians so all study arms will receive the same standard counseling and clinical procedures. The determination of the primary outcomes will be based on objective measures of heavy alcohol drinking and adherence and are thus unlikely to be subjected to bias. The statistician will be blinded to the intervention group allocation of participants during the analysis stage.

## Data collection and management

Data entry, data management, and quality assurance are conducted at the four study sites. However, all standard operating procedures for study assessment and data collection are identical across the study sites. The data management team will monitor data quality and track and link the multiple data sources. The team has jointly developed the data collection forms, designed the database management system for data entry and for participant tracking, and implemented procedures for quality control, and they will collaborate on report writing and the presentation of study results.

Data will be collected and recorded by research assistants using password-protected laptop computers. Electronic data collection forms provide clear instructions to guide data entry and include preprogrammed skip patterns, real-time range checks, and internal logic to minimize missing data. The questionnaire data, assessment of side effects at refill visits, and laboratory results are entered into laptop computers in Uganda while offline and later uploaded to a secure server at the University of California San Francisco (UCSF). The MEMS adherence data are uploaded at least monthly. Hair levels are analyzed in the UCSF Hair Analytical Laboratory (HAL) and the data will be uploaded locally. Tracking information is entered into a database that generates reminders for all follow-up visits. The databases are located on secure, password-protected servers in Uganda and the USA.

At each site, all data will undergo weekly checks for completeness and range criteria, including multiple checks for entered data. Electronic data will be collected using password-protected laptop computers, and the study database will be located on secure, password-protected servers in Uganda and the USA.

## Statistical analysis

For the primary outcomes, we will estimate the intention-to-treat effect by including all participants according to their randomized intervention. We will compute descriptive statistics on baseline variables overall and by randomization group to characterize the study population and to detect any baseline imbalance. For continuous measures, we will report the mean, standard deviation, median, interquartile range, and the minimum and maximum. For categorical data, we will report counts and percentages.

For Aim 1, we will use multiple logistic regression models to determine the proportion of participants with no heavy drinking during INH where the main independent variable is a binary indicator representing assignment to financial incentives for decreasing alcohol use (yes versus no) while adjusting for incentives for INH adherence. Similarly, for Aim 2, we will use a multiple logistic regression model to assess the proportion achieving high INH adherence where the main independent variable is a binary indicator representing assignment to financial incentives for increasing adherence to INH (yes versus no) while adjusting for incentives for alcohol reduction. All models will be adjusted for the randomization stratification factors (gender and study cohort) to improve efficiency. We will also adjust for baseline characteristics that differ by study arm despite randomization.

We will use similar methods for other binary secondary outcomes. We will fit linear regression models to examine continuous secondary outcomes including PEth, hair levels of INH, and percentage doses taken over 6 months based on MEMS. We will explore transformations for variables that are not normally distributed. If an appropriate transformation is not identified, median regression models will be used.

We will use multiple logistic regression models to assess the proportion of participants with HIV virologic suppression at the 12-month visit (a secondary outcome) where the main independent variable will be a categorical variable for the assigned incentive intervention. We will conduct three pairwise comparisons to estimate the effect of each of the three separate incentive interventions (Arms 2, 3, and 4) versus no incentives (Arm 1) and will adjust for multiple comparisons using the Hochberg sequential test procedure [65] to keep the overall family-wise error at 0.05.

Mediation analysis methods will explore the role of alcohol use and level of IPT adherence as potential mediators that may drive the interventions to improve HIV virologic suppression. More specifically, we will use methods for mediation analysis to derive the direct and indirect effects of the incentives for the primary outcomes while allowing for exposure-mediator interactions [66–72].

As exploratory analyses, for Aims 1 and 2, we will check for the presence of intervention-by-time interaction by conducting separate analyses at months 3 and 6. We will use generalized estimating equations with an independent working correlation matrix for binary outcomes and random effect models for continuous outcomes. In case of missing data, we will employ a number of methods including multiple imputations to assess the sensitivity of the results. Finally, we will conduct subgroup analyses to explore whether sex, baseline alcohol use, readiness to change, and discount rate act as effect modifiers between financial incentives and the primary outcomes.

No interim efficacy or futility analyses are planned because the primary risk, hepatotoxicity, will be examined by the investigators and the data and safety monitoring board on an ongoing basis.

We will follow the CONSORT statement guidelines in reporting. We will use the statistical software STATA to carry out the analyses and *P*-value <0.05 will be considered as statistically significant.

## Monitoring

The study team, including the project directors, statistician, co-investigators, field coordinators, and research assistants, led by the Principal Investigators, will monitor the progress of the study, participant recruitment, accrual, and retention at twice monthly conference call meetings. All adverse events (AE) will be discussed. They will also examine factors external to the study when interpreting the data, such as scientific developments or the new availability of information that could impact the safety of the participants, such as new pertinent FDA Drug and Safety Announcements, the performance of the study, or the ethics of the study.

To ensure the safety of the participants, the integrity of the data, and quality and scientific validity of the study, all safety data are reviewed every 6 months by an independent data safety monitoring board (DSMB) external to the study team and the sponsor (National Institute of Health). Specifically, the DSMB reviews the plans and processes for identifying individual or patterns of adverse events and review accumulating safety data. Following each meeting, the DSMB makes recommendations on the continuation, modification, or termination of the study.

Day-to-day data and safety monitoring will be the responsibility of the Principal Investigators and will follow guidelines set forth by the IRB regarding AEs. AEs will be reported to the IRBs within 10 days of awareness of the event. AEs will be monitored for each subject participating in the study and attributed to the study intervention by the Principal Investigator with review by the physicians/co-investigators according to the following categories: definite, probable, possible, unlikely, and unrelated. Serious adverse events (SAEs) will also be reported to IRB as well as to the National Health Institute (NIH) within 2 days. An annual report of all AEs will be submitted to the National Institute of Alcohol Abuse and Alcoholism (NIAAA).

## Qualitative study to examine potential pathways of intervention action

As a complement to the trial, we will conduct a cross-sectional qualitative study consisting of individual, in-depth, semi-structured interviews with study participants and family members to gain an in-depth understanding of how and why financial incentives are instrumental in promoting a decrease in alcohol use and INH adherence (or why not, if they fail to do so in some cases or settings). We also aim to identify any potential collateral impacts on or changes in health behaviors that are complementary to the main study outcomes, including HIV risk behavior, ART adherence, financial well-being, interpersonal relationship dynamics including intimate partner and domestic violence, and health protective behaviors. An initial sample of 32 study participants completing their 6-month visit will be systematically selected for recruitment from the DIPT study population, from randomly generated lists of participants within the strata of study site, study arm, and gender (with a target of 25% women, reflective of the population of HIV/TB infected who engage in heavy drinking). The selected participants will be asked to invite either a spouse or a close family member, who is aware of the participant’s study activities, to engage in a separate in-depth interview. We will interview spouses/family members separately, with the goal of recruiting a sample of at least 16, but up to 32 spouses/family members, stratified to be composed of approximately even proportions across study sites and arms as well as gender. Consistent with theoretical sampling approaches [73], if we determine during the initial data analysis phase that data have not attained an adequate level of theoretical saturation with the above samples, up to 16 additional study participants may be selected according to theoretically salient categories as needed, time and resources permitting.

The in-depth interviews will be conducted by a Ugandan research assistant (RA) trained in qualitative research methods, who will pursue pre-determined lines of questioning to ensure both that all topics are covered allowing participants to discuss topics salient to the study and that the data can be effectively analyzed. The interviews will be audio-recorded, transcribed, and then translated into English by the RA jointly with a transcriptionist. The RA will also produce brief summary notes on the same day in which the data are collected. The interview guide will be designed to elicit participants’ attitudes, beliefs about, and experiences with heavy drinking and INH and ART adherence, including an exploration of the barriers and facilitators of behaviors, and how these may have changed during the intervention. We will explore attributions for perceived change, including questions about the study visits and incentives, and how they affected (or did not affect) the outcomes. We will explore how the incentives were perceived by their partners and family members, whether and how the incentives affected relationship quality, and participants’ alcohol use and INH and ART adherence. We will allow for open exploration of additional mediators of change related to the incentives.

A second area of inquiry will examine whether and how beliefs, attitudes, and experiences related to secondary social/behavioral outcomes, including sexual risk behavior, domestic violence, employment and savings gains, social standing, and appointment keeping, were impacted by the intervention. We will explore whether and how anything else in their life (or their partner’s life) has changed that they attribute to the intervention experience. We will also explore whether and how secondary benefits (when they occurred) fed back into motivation and the ability to reduce alcohol use and maintain medication adherence. We will further explore any unintended consequences or unforeseen other positive or negative effects of the intervention on the participants’ lives.

Interview transcripts and notes will be analyzed using a hybrid deductive and inductive analysis method informed by constructivist grounded theory [73] and a systematic interpretivist approach in the domain of theory-generative research [74], which involves both “abductive” and “inductive” analyses of empirical data and production of findings, to foster theory construction and generate new explanatory hypotheses. In this method, the study team will iteratively code and analyze the data on the basis of an a priori theory-based coding framework, which is subsequently adapted and refined at defined intervals on the basis of empirical findings. We will use a qualitative analysis software program for textual analysis that facilitates team-based collaborative coding, to assist with organizing the data. An initial coding framework will be developed by the lead investigator and iteratively refined at defined periods during data collection by the investigators and field team. The team will also discuss and resolve divergent coding and interpretations of findings as a group, both to ensure consistency (inter-coder reliability) and to review unexpected but salient findings as they emerge in the data. Contradictions, inconsistencies, and deviant cases in the data will be discussed and notated in the analysis process. At the final stage of analysis, analytical memoranda will be produced outlining the emergent themes with supportive evidence to characterize pathways of intervention action, with attention to contradictory evidence and deviant cases.

## Discussion

The DIPT 2×2 factorial randomized controlled trial will examine the effect of financial incentives to decrease alcohol consumption and to increase INH adherence and HIV virologic suppression among PWH with TB co-infection who engage in heavy alcohol drinking in Uganda. This trial will contribute to the identification of effective interventions to facilitate TB prevention in a very high-risk population and is, therefore, well-aligned with the WHO End TB guidance calling for treatment for all people with TB [75].

Financial incentives have been effective in promoting behavior change in various health domains. The targeted behaviors have included reductions in substance use [76] and HIV prevention behaviors such as HIV testing [77] and medical male circumcision [78]. Options for reducing alcohol use and preventing TB are currently lacking for PWH who engage in heavy alcohol drinking in resource-limited settings [79]. Indeed, PWH with heavy drinking are often excluded from receiving IPT. If proven effective, our interventions will offer a potentially scalable tool for reducing heavy alcohol use, preventing TB, and possibly improving ART adherence in a subset of PWH at high risk for poor outcomes in Uganda and other settings with high prevalence of HIV and TB.

The study will leverage the availability of newly available objective, POC tests for alcohol use and INH adherence in resource-limited settings. These tests allow for the implementation of financial incentive interventions conditioned on and aimed at promoting reductions in alcohol use and adherence to INH. The work we describe has already launched and we are in the first year of successful recruitment and follow-up, suggesting that the use of the POC tests and the intervention are feasible in clinical practice and acceptable to participants.

In low-income settings, INH is typically not recommended for persons with latent TB who engage in heavy drinking due to the high risk of liver toxicity in this population [80]. The high risk of hepatotoxicity is exacerbated by the lack of availability of routine laboratory tests for liver enzymes (AST and ALT) [81]. Because of this, little is known on the frequency and severity of side effects and on the level of adherence to INH in this important group. The DIPT trial will provide an opportunity to characterize side effects of INH, while ensuring patient safety through monthly monitoring for liver enzymes. However, our control group will be screened for side effects more intensively than in the standard of care in Uganda, particularly because most side effects are expected to be mild and reversible and typically, not detectable without liver enzyme monitoring.

The study has some limitations. First, the study is not double blinded and individuals randomized to different groups might also have different expectations that affect their participation in the trial. Second, because individuals with liver toxicity or inebriation at screening are not eligible, our study population might be more likely to exclude people with very severe drinking. Finally, new TB preventive therapy regimens (e.g., using combinations of INH and rifapentine or rifampin monotherapy) of shorter duration than IPT might offer advantages in reducing duration of treatment and improving adherence, but they are not being assessed in this study. However, the risk of INH and alcohol-related hepatotoxicity suggests a future utility of this intervention for the other regimens.

## Trial status

Protocol version 1.5, May 2, 2018. Recruitment is ongoing. Recruitment started on April 16, 2018. As of August 18, 2020, 3520 individuals have screened for eligibility at the study sites and 485 were enrolled into the trial and randomized. A total of 316 individuals have completed their INH course and 262 have attended the 12-month visit. The qualitative study started in June 2020. The trial is registered under protocol number NCT03492216 in clinicaltrials.gov.

Study activities were paused on March 19, 2020, due to a lockdown enforced by the Ugandan government in response to the COVID-19 pandemic. During this time, all research activities were stopped and participants on INH were contacted and instructed to stop taking their INH pills immediately, as there were no means to effectively monitor for liver toxicity. Study visits resumed on May 26, 2020, and enrollment resumed on June 16, 2020. Due to the COVID-19 pandemic, the anticipated end of recruitment date, February 28, 2021, has been postponed. We now expect to enroll the target sample size in August 2021 and plan to conclude follow-up in August 2022.

## Data Availability

Data will be available first to DIPT investigators and trainees, followed by URBAN ARCH Consortium investigators and trainees. After establishing the specific areas to be addressed in the papers reporting the main findings of the study, the data will be made available in a reasonable timeframe to interested and qualified researchers wishing to conduct secondary analyses of the data. Priority will be given to investigators that address alcohol, HIV, and TB research issues. To facilitate follow-up, we will be collecting identifying information during the trial. However, the final datasets will be stripped of identifiers prior to release for sharing. Research results will be published in international indexed journals. No individual identities will be used in any reports or publications resulting from the study.

## Abbreviations

TB: Tuberculosis
HIV: Human immunodeficiency virus
PWH: Person living with HIV
INH: Isoniazid
IPT: Isoniazid preventive treatment
ART: Antiretroviral therapy
DIPT: Drinkers’ Intervention to Prevent Tuberculosis
POC: Point of care
EtG: Ethyl glucuronide
AUDIT-C: Alcohol Use Disorder Identifier Test-Consumption
TST: Tuberculin skin test
AST: Aspartate aminotransferase
ALT: Alanine aminotransferase
PEth: Phosphatidylethanol
MEMS: Medication Event Monitoring System
DBS: Dried blood spots
